# Successful amnioinfusion for severe fetal growth restriction with umbilical cord complications: two case reports

**DOI:** 10.1186/s13256-021-02904-4

**Published:** 2021-07-29

**Authors:** Daisuke Katsura, Yuichiro Takahashi, Shigenori Iwagaki, Rika Chiaki, Kazuhiko Asai, Masako Koike, Shunichiro Tsuji, Fuminori Kimura, Takashi Murakami

**Affiliations:** 1 Department of Obstetrics and Gynecology, Shiga University of Medical Science Hospital, Otsu, 520-2192 Japan; 2 Department of Fetal-Maternal Medicine, Nagara Medical Center, 1300-7, Nagara, Gifu, 502-8558 Japan

**Keywords:** Amnioinfusion, Fetal growth restriction, Oligohydramnios, Umbilical cord compression, Umbilical cord factors

## Abstract

**Background:**

There is no established treatment for fetal growth restriction during pregnancy. We report two cases that represent an example of an amnioinfusion-based management strategy for severe fetal growth restriction with umbilical cord complications.

**Case presentation:**

We encountered two cases of fetal growth restriction with abnormal fetal Doppler velocity. In one case, fetal ultrasound revealed a hypercoiled umbilical cord with a single umbilical artery and oligohydramnios, while fetal Doppler revealed a reversed end-diastolic flow in the umbilical artery and reversed a-waves of the ductus venosus. Umbilical cord compression was confirmed at 22 weeks and 2 days of gestation, and nine amnioinfusions were performed to relieve the umbilical cord compression. A cesarean section was performed at 31 weeks and 2 days of gestation because of severe preeclampsia. The Asian infant is now a normally developed 6-month-old. In another Asian case, fetal ultrasound revealed a hypercoiled cord, while fetal Doppler revealed a reversed end-diastolic flow in the umbilical artery and intermittent reversed a-waves of the ductus venosus. Umbilical cord compression was confirmed at 24 weeks and 5 days of gestation, and seven amnioinfusions were performed. A cesarean section was performed at 31 weeks and 1 day of gestation because of nonreassuring fetal status. At the age of 1 month, the Asian infant was stable on respiratory circulation. In both cases, fetal Doppler findings improved significantly following amnioinfusions.

**Conclusions:**

Amnioinfusion is a symptomatic treatment for umbilical cord compression. However, to determine the therapeutic effect of amnioinfusion, complete resolution of the umbilical cord compression should be ascertained by ultrasonography.

## Background

Fetal growth restriction (FGR) is associated with neonatal morbidity and mortality, and requires intensive management [1]. FGR with oligohydramnios and abnormal fetal Doppler velocity are associated with severe placental insufficiency and perinatal mortality [2–4]. However, there is no established treatment for FGR during pregnancy. Therefore, delivery is considered depending on the gestational age and the estimated fetal weight (EFW).

Amnioinfusion (AI) is effective for treating variable decelerations in the fetal heart rate caused by umbilical cord compression with oligohydramnios [5, 6]. AI has also been reported to improve abnormal fetal Doppler velocity in cases of FGR with oligohydramnios [4, 7, 8] and in those with umbilical cord compression without oligohydramnios [9]. However, the guidelines for managing FGR and umbilical cord compression using AI are unclear. Herein, we report two cases of patients with extremely severe FGR with umbilical cord complications and abnormal fetal Doppler velocity, in whom AI was useful for prolonging gestation. These cases may present an example of an AI-based management strategy for severe FGR with umbilical cord complications.

## Case presentation

### Case 1

A 33-year-old Asian woman (gravida 1, para 0) was referred and admitted to our hospital for the management of FGR and oligohydramnios at 22 weeks and 2 days of gestation. She had become pregnant via *in vitro* fertilization and had been followed up routinely by her obstetrician. She did not complain of water discharge. Fetal ultrasound screening revealed that the EFW, maximum vertical pocket, and coiling index were 204 g [−3.7 standard deviation (SD)], 1.8 cm, and 0.76, respectively. FGR, oligohydramnios, hypercoiled umbilical cord, and a single umbilical artery were detected. Fetal structural anomalies were not detected. On fetal Doppler, reversed end-diastolic flow in the umbilical artery (UA), reversed a-waves of the ductus venosus (DV), and pulsatile umbilical venous flow were detected. Cardiotocography (CTG) revealed that the fetal heart rate at baseline was 150 beats per minute with no variability, and recurrent decelerations were noted. Fetal death was imminent as there was umbilical cord compression caused by oligohydramnios and umbilical cord factors. Therefore, AI was performed for relieving cord compression. Under ultrasonographic guidance, a 21-gauge percutaneous transhepatic cholangiography needle was introduced into the amniotic cavity, and 250 mL of warm saline was infused to achieve a normal amniotic fluid volume, as defined by an amniotic fluid index (AFI) of 12 cm.

**Fig. 1.**
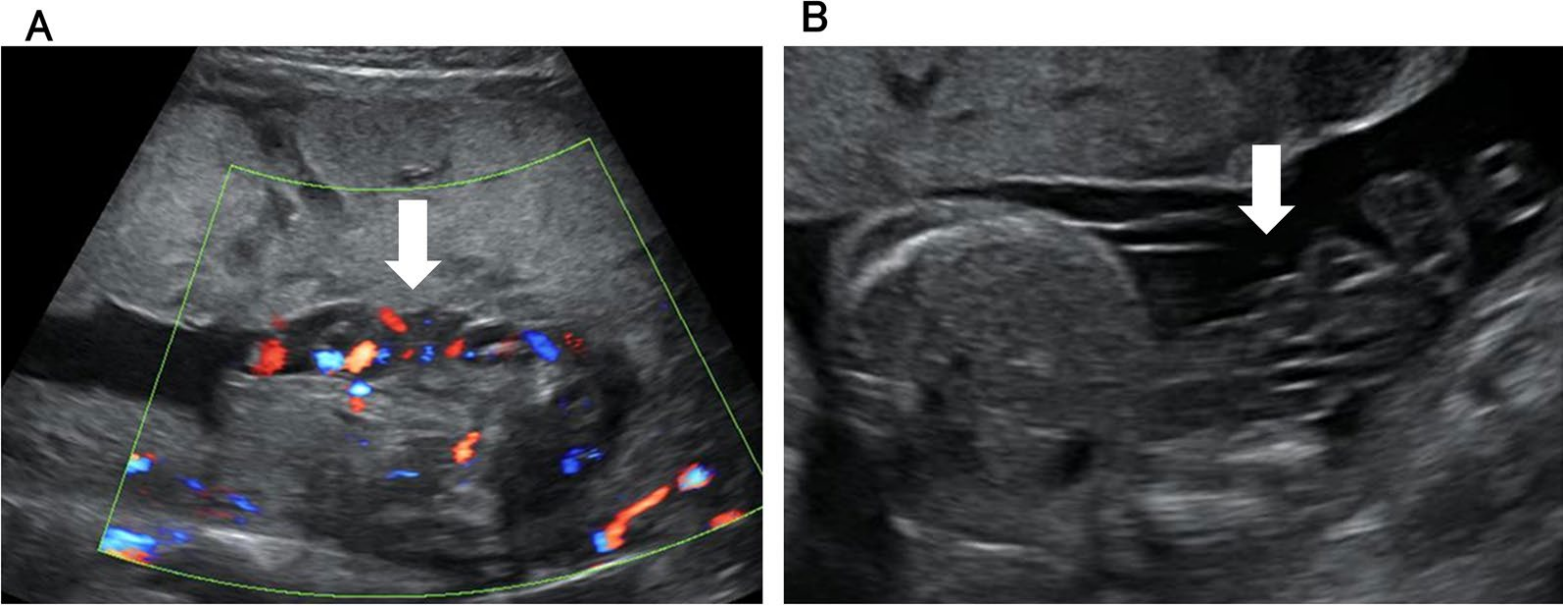
Fetal ultrasonography findings in case 1. **A ** The “sandwich sign,” that is, the umbilical cord sandwiched between the placenta and the fetus, is observed (white arrow). **B** The umbilical cord compression has resolved (white arrow)

Two days later, that is, at 22 weeks and 4 days of gestation, we observed an intermittent reversed end-diastolic flow in the UA and a-waves of the DV on fetal Doppler. Therefore, we believed that AI was effective, and performed another AI as we observed that the umbilical cord was compressed between the placenta and the fetus (Fig. 1A), although oligohydramnios was not detected (AFI: 11 cm). Two days later, that is, at 22 weeks and 6 days of gestation, a decreased baseline variability of the fetal heart rate, with no decelerations, was observed. Fetal Doppler showed a UA pulsatility index (PI) of 1.50, middle cerebral artery PI of 0.88, and DV-PI of 1.01. While the brain-sparing effect (cerebroplacental Doppler ratio 0.58) and a high DV-PI were noted, both CTG and fetal Doppler findings had improved. Therefore, when umbilical cord compression was confirmed by the detection of a sandwiched umbilical cord during ultrasonography and reproducible variable decelerations on CTG, we repeated AI. At 25 weeks and 1 day of gestation, the patient was diagnosed with severe hypertensive disorders of pregnancy and was administered a calcium blocker. At 26 weeks and 2 days of gestation, the umbilical cord compression was found to have resolved on ultrasonography (Fig. 1B), and CTG showed normal baseline variability with accelerations. In total, nine AIs were performed.

A maternal urine protein level of 2 g per day was confirmed; therefore, corticosteroids and magnesium sulfate were administered for fetal lung maturation and neuroprotection, respectively. Magnesium sulfate was administered until delivery. A cesarean section was performed at 31 weeks and 2 days of gestation because of severe preeclampsia. A female infant, weighing 870 g, was born with Apgar scores of 8 at 1 minute and 8 at 5 minutes. Her umbilical arterial blood pH was 7.345, and she was admitted to the neonatal intensive care unit (NICU) because of the extremely low birth weight. In the NICU, she remained stable with directional positive airway pressure, and was not diagnosed with the respiratory distress syndrome. She did not develop intraventricular hemorrhage, retinopathy of prematurity, chronic lung disease, or necrotizing enterocolitis. She was discharged home at 41 weeks and 6 days of adjusted gestational age, and weighed 2300 g at the time of discharge. She is being followed up as an outpatient, and is presently a normally developed 6-month-old infant.

### Case 2

**Fig. 2.**
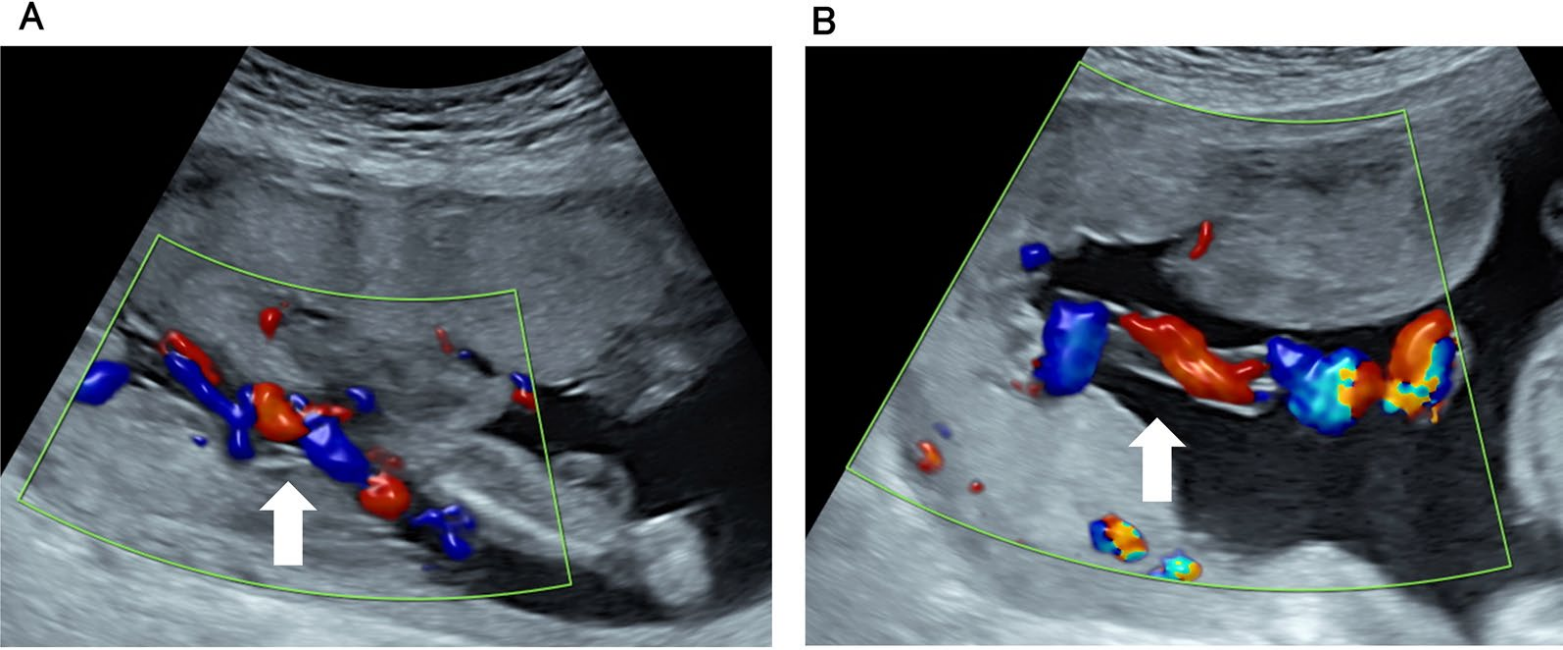
Fetal ultrasonography findings in case 2. **A** The “sandwich sign,” that is, the umbilical cord sandwiched between the placenta and the fetus, is observed (white arrow). **B** The umbilical cord compression has resolved (white arrow)

A 33-year-old Asian woman (gravida 3, para 1) was admitted to our hospital for the management of FGR with an abnormal fetal Doppler velocity at 24 weeks and 5 days of gestation. Owing to antithrombin deficiency, she was being administered heparin (10,000 units per day subcutaneously) and was infused with Neurt (3000 units, three times per week). Fetal ultrasound screening revealed that the EFW, AFI, and coiling index were 376 g (−3.2 SD), 12 cm, and 0.75, respectively. FGR and a hypercoiled umbilical cord were detected. Fetal structural anomalies were not detected. On fetal Doppler, reversed end-diastolic flow in the UA, intermittent reversed a-waves of the DV, and pulsatile umbilical venous flow were detected. Corticosteroids and magnesium sulfate were administered for fetal lung maturation and neuroprotection, respectively. Magnesium sulfate was administered for 2 days. We observed that the umbilical cord was compressed between the placenta and the fetus, although oligohydramnios was not detected (Fig. 2A). Therefore, we performed AI for relieving umbilical cord compression, because it was considered to be an underlying cause of the deterioration of fetal condition. After the umbilical cord compression was found to be resolved by AI on ultrasonography (Fig. 2B), we observed an UA-PI of 1.84, MCA-PI of 1.19, and DV-PI of 1.08. Though a brain-sparing effect (cerebroplacental Doppler ratio 0.64) and a high DV-PI were noted, the fetal Doppler findings were improved. This suggested that AI was effective. Therefore, we repeated AI when umbilical cord compression was confirmed by the detection of a sandwiched umbilical cord during ultrasonography and reproducible variable decelerations during CTG. In total, seven AIs were performed.

At 31 weeks and 1 day of gestation, we observed reversed end-diastolic flow in the UA and intermittent reversed waves of the DV on fetal Doppler, as well as frequent variable decelerations on CTG; however, umbilical cord compression was not confirmed. Therefore, a cesarean section was performed because of nonreassuring fetal status. A male infant, weighing 760 g, was born with Apgar scores of 4 at 1 minute and 9 at 5 minutes. His umbilical arterial blood pH was 7.308, and he was admitted to the NICU because of the extremely low birth weight. In the NICU, he was managed with directional positive airway pressure and was extubated at 12 days of age. At the age of 1 month, he remained stable on respiratory circulation.

## Discussion

Herein, we have reported the cases of two patients with extremely severe FGR with umbilical cord complications and abnormal fetal Doppler velocity, in whom AI was useful for prolonging gestation. Although fetal death was imminent, we could not deliver the patients because of the gestational age and the EFW, as the infants would not have survived. We believe that the umbilical cord blood-flow disturbance, which was caused by umbilical cord compression secondary to entire or local oligohydramnios arising from umbilical cord complications (such as a hypercoiled cord and a single umbilical artery), contributed to the severe condition of the patients. Takahashi *et al*. and Iwagaki *et al*. reported an improvement in abnormal UA and DV Doppler findings after administration of AI in cases with FGR with oligohydramnios and umbilical cord complications [4, 8]. Repetitive umbilical cord compression induces fetal asphyxia [4, 10], and AI relieves umbilical cord compression, decreases the vascular resistance of UA, increases umbilical venous blood flow, and improves oxygenation and the intrauterine environment [9]. However, the efficacy of AI has not yet been established. Therefore, we informed these patients about AI and the associated complications before obtaining their consent. Our findings were similar to those of a previous study [9], wherein the sandwich sign, indicative of the umbilical cord being sandwiched between the placenta and the fetus resulting in cord compression, was detected on ultrasonography, although oligohydramnios had resolved. In case 1, the initial AI improved the fetus’s well-being slightly, but the second AI in case 1 and the initial AI in case 2, which relieved the umbilical compression, markedly improved the intrauterine conditions. We believe that the umbilical cord blood-flow disturbance was one of the causes of fetal complications, and complete resolution of the umbilical cord compression was necessary. Hence, repeated AIs led to improved CTG and fetal Doppler findings, which allowed for longer pregnancies, resulting in the survival of the infants without sequelae.

AI is not a suitable treatment for the following factors: (1) umbilical factors such as a hypercoiled umbilical cord and an abnormal umbilical cord insertion, (2) placental factors such as hypertensive disorders of pregnancy and massive subchorionic hematomas, and (3) fetal factors such as chromosomal abnormalities, congenital anomalies, and infections. Instead, it is a treatment for umbilical cord blood-flow disturbances caused by umbilical cord compression secondary to entire or local oligohydramnios that is caused by these factors [4, 9]. Case 1 was associated with umbilical and placental factors, as hypertensive disorders of pregnancy developed and led to the delivery of the infant. Case 2 was associated with umbilical cord blood-flow disturbance as an umbilical factor secondary to a hypercoiled umbilical cord, which deteriorated and led to the delivery of the infant. Therefore, AI is a symptomatic treatment for umbilical cord compression; it is not an accurate treatment for FGR.

Adverse events related to AI have been reported, including premature rupture of membranes, preterm labor, placental abruption, chorioamnionitis, fetal trauma, and uterine perforation [11]; therefore, AI should only be performed in carefully selected patients by well-trained experts. In cases with umbilical cord compression secondary to local oligohydramnios, changes in the maternal position or fetal movement might relieve the compression. Therefore, we considered reproducible variable decelerations on CTG as the indication for AI. The efficacy of AI has not yet been established, and though AI may cause some complications, there are no detailed studies on the complications of AI. Therefore, further studies are needed to clarify the efficacy of AI.

In addition, we generally undertake CTG examinations from 26 weeks of gestation, because the developmental state of the fetal autonomic nervous system must be considered for the interpretation of CTG findings [12], and there is limited knowledge on the CTG findings regarding FGR in fetuses at less than 26 weeks of gestation [13]. In our cases, although the gestational age was less than 26 weeks, we performed CTG to confirm the absence of umbilical cord compression, because umbilical cord compression causes variable deceleration on CTG [9, 14].

## Conclusion

Although amnioinfusion is a symptomatic treatment for umbilical cord compression, it was a life-saving therapeutic strategy for the patients described in this report. However, to determine the therapeutic effect of amnioinfusion, complete resolution of the sandwich sign, which indicates umbilical cord compression in cases of FGR with umbilical cord complications, must be ascertained by ultrasonography.

## Data Availability

Not applicable.

## Abbreviations

AFI: Amniotic fluid index
AI: Amnioinfusion
CTG: Cardiotocography
DV: Ductus venosus
EFW: Estimated fetal weight
FGR: Fetal growth restriction
NICU: Neonatal intensive care unit
PI: Pulsatility index
SD: Standard deviation
UA: Umbilical artery
